# The links between supplementary tannin levels and conjugated linoleic acid (CLA) formation in ruminants: A systematic review and meta-analysis

**DOI:** 10.1371/journal.pone.0216187

**Published:** 2020-03-13

**Authors:** Rayudika Aprilia Patindra Purba, Pramote Paengkoum, Siwaporn Paengkoum

**Affiliations:** 1 School of Animal Technology and Innovation, Institute of Agricultural Technology, Suranaree University of Technology, Nakhon Ratchasima, Thailand; 2 Program in Agriculture, Faculty of Science and Technology, Nakhon Ratchasima Rajabhat University, Nakhon Ratchasima, Thailand; University of Agriculture in Krakow, POLAND

## Abstract

A systematic review and meta-analysis were conducted to predict and identify ways to increase conjugated linoleic acid (CLA) formation in ruminant-derived products to treat human health issues with dietary tannins. The objective was to compare and confirm the effects of dietary tannins on CLA formation by analyzing *in vitro* and/or *in vivo* studies. We reported the results of the meta-analysis based on numerical data from 38 selected publications consisting of 3712 treatments. Generally, via multiple pathways, the CLA formation increased when dietary tannins increased. Concurrently, dietary tannins increased Δ^9^ desaturation and the CLA indices in milk and meat (*P* < 0.05 and *P* < 0.001, with average R^2^ values of 0.23 and 0.44, respectively), but they did not change the rumen fermentation characteristics, including total volatile fatty acids (mmol/L) and their acid components. *In vitro* observations may accurately predict *in vivo* results. Unfortunately, there was no relationship between *in vitro* observations and *in vivo* results (R^2^ < 0.10), indicating that it is difficult to predict CLA formation *in vivo* considering *in vitro* observations. According to the statistical meta-analysis results regarding animal aspects, the ranges of tannin levels required for CLA formation *in vitro* and *in vivo* were approximately 0.1–20 g/kg dry matter (DM) (*P* < 0.001) and 2.1–80 g/kg DM (*P* < 0.001), respectively. In conclusion, the *in vivo* method was more suitable for the direct observation of fatty acid transformation than the *in vitro* method.

## Introduction

There is a substantial demand for ruminant-derived products, such as milk and meat, and quality, especially fat content, has become increasingly important to consumers recently [1]. Saturated fatty acids (SFAs) are present in higher concentrations than polyunsaturated fatty acids (PUFAs) in milk and meat [2]. The relationship between dietary SFAs and the risk of coronary heart diseases (CHDs) is strong; CHDs result in 2155 coronary-related deaths among 344,696 persons annually and are caused by SFA accumulation in the human body when energy intake is unbalanced, resulting in unhealthy levels of low-density lipoprotein (LDL) and high-density lipoprotein (HDL) cholesterol [3]. Habitual alteration may result in a “healthy life” by reducing SFA intake by approximately <10%, relative to total energy and cholesterol intake, to <300 mg/d [4]. Over the past 2 decades, many nutritionists around the world have conducted relevant studies to solve this problem and have identified a natural PUFA with conjugated linoleic acid (CLA) in milk and meat as a natural key element. Most of the researchers found that CLA has a health-promoting value in humans concerning the prevention of CHDs, abating or eliminating cancer, improving immunity and treating obesity through the development of fixed lean body mass [5–7].

Despite the considerable benefits of CLA, it can be hydrolyzed and is present in limited ranges of 0.34–1.07% of total fat in milk and 0.12–0.68% of total fat in meat [8]. Further, CLA is an intermediate product produced by shortening linoleic acid (LA; cis-9, cis-12 18:2) and α-linolenic acid (ALA; cis-9, cis-12, cis-15 18:3) through lipolysis, isomerization, and biohydrogenation; the dominant isomers of CLAs are cis-9, trans-11 18:2 and trans-7, cis-9, representing 75–90% and 3–16% of the total CLAs, respectively [2, 9]. Unfortunately, ruminants do not have the ability to endogenously synthesize LA and ALA [10]. Two possible suggestions by Chilliard, et al [11] to obtain PUFAs, including CLAs, in ruminant-derived products are as follows: an altered biohydrogenation pathway with regard to microorganism reductase and the origin of the diet in the feeding regimen. It is well documented that bacteria from group A, *Butyrivibrio fibrisolvens*, which transforms LA and ALA to cis-9, trans-11 18:2 and trans-11 18:1 isomers [2], is active in the first stage of biohydrogenation. Additionally, an activated enzyme remove two hydrogen atoms with Δ^9^ desaturase through desaturation deposit more cis-9, trans-11 18:2 isomers [8, 12, 13]. There has been a particular interest in comparing the roles of other microorganisms in biohydrogenation, namely, protozoa and fungi. Protozoa contribute to 30–40% of lipolysis activity by adhering to the plant surface (possibly in feedstuff) by an anterior pleated zone, resulting in an efficient breakdown of cellular membranes [14]. As a consequence, rumen protozoa were confirmed to be a rich source of PUFAs due to the ingestion of more chloroplasts [15]. Likewise, rumen protozoa of *Epidinium* spp. were reported to have a positive association (*P* < 0.05) with cis-9, trans-11 18:2 and trans-11 18:1 depositions [16]. However, *Epidinium* spp. were reported to be unable to induce Δ^9^ desaturation [15, 17] associated with bacterial lipids. Rumen fungi were represented by the *Orpinomyces* genus, which also accelerated CLA production via the LA lipolysis isomerization stage in slow catalysis. However, the double-bonded reductase of the ALA fraction was unclear [18].

The original diet in the feeding regimen can be supplemented with feeds or additional fats, such as forages, animal lipids and vegetable oils, to achieve increased CLA fractions in milk and meat, as reported in a previous meta-analysis [19]. Recently, the efficiency of the aforementioned supplementation was confirmed by its affinity for bacterial lipid from group A, which is toxic to microorganism [2]. Notably, microorganisms that interrupt lipid production seem to have a clear association with the change in the fatty acid (FA) profiles in milk and meat. Moreover, tannins applied as phytochemicals in feeding regimens have been shown to have antimicrobial properties. Typical forms of condensed and hydrolysable tannins have been completely tested to obtain information regarding the bioactive role of target plants on rumen fermentation considering biohydrogenation [20, 21]. Additionally, tannins promoted group B of bacteria (*Butyrivibrio proteoclasticus*), which convert trans-11, C18:1 forms to C18:0 forms [22]. In other words, tannins could be supplemented in feed to improve CLA production via the inhibition of C18:0 (SA: stearic acid), resulting in increased Δ^9^ desaturation.

Furthermore, two prior meta-analyses showed that dietary nutrients containing PUFAs and tannins had a dependent relationship with feeding type [23, 24]. Grazing regimens tended to promote cis-9, trans-11 18:2 and trans-11 18:1 (VA: vaccenic acid) depositions in ruminant-derived products. There was also a systematic literature review and meta- and redundancy analyses [25] that discussed organic provisions for the alteration of biohydrogenation, resulting in expected increases in CLA deposition in milk. The abovementioned publications focus on valuable tannins, edible applications and potential functions separately. However, none of the studies addressed the relationship between tannin supplementation in ruminant diets and biohydrogenation with the meta-analysis technique. A clear-cut method, whether using *in vitro* or *in vivo* methods, to obtain baseline data is needed. Hopefully, the results of the present meta-analysis will contribute to animal science, animal nutrition and biotechnology knowledge. The objectives of this study were (i) to elucidate the effectiveness of tannins in the modulation of CLA formation, (ii) to compare the results of *in vitro* and *in vivo* methods, and (iii) to confirm the relationship between *in vitro* and *in vivo* studies, applying meta-analysis as a statistical tool.

## Methods

### Search strategy and selection criteria

**Fig 1:**
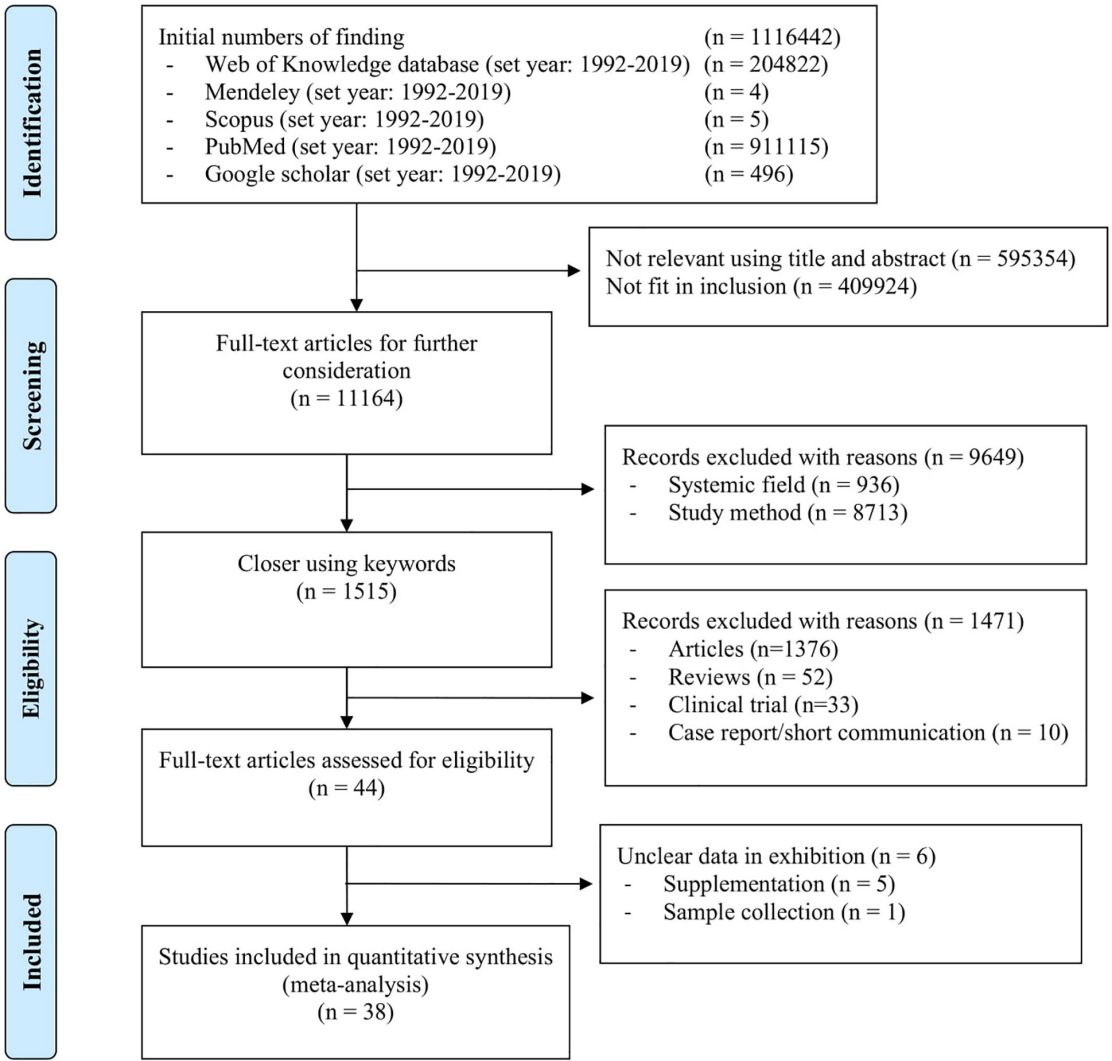
Literature retrieval flow chart.

A database of previous studies involving dietary tannins and CLA properties was created considering the Preferred Reporting Items for Systematic Reviews and Meta-Analyses (PRISMA) guidelines (Fig 1) [26]. These publications were gathered from the Web of Knowledge, Mendeley, Scopus, PubMed and Google Scholar databases. The following keywords were applied in each database search (S1 Table): “biohydrogenation,” “conjugated linoleic acid,” “rumen,” “tannin,” “condensed tannin,” “hydrolysable tannin,” “meat,” “milk,” “*in vivo*” and “*in vitro*.” The databases were searched from January 1992 to March 2019, resulting in 1116442 references. The papers were published in all languages in peer-reviewed and non-peer-reviewed journals and comprised single articles, review articles, clinical trials and case report/short communications. Relevant papers were deposited into and duplicate papers were removed by Endnote (Thompson ISI Research-Soft, Philadelphia, PA, US).

### Study criteria, quality assessment and data extraction

The study criteria, quality assessment and data extraction processes followed those in a previous meta-analysis [24, 25]. The inclusion criteria were as follows: (1) the study utilized an *in vitro* method; (2) the study utilized an *in vivo* method; (3) the study subjects were ruminants, including cows, goats, and sheep that produced dairy or meat products; (4) the study contained relevant data that was retrievable; and (5) the study was published after 1 January 1992. Authors were contacted by e-mail and through ResearchGate if the data was questionable. If contact was unsuccessful, the studies were excluded because of the inaccessibility of the data.

All the numerical data from the text, tables and figures of screened papers were copied directly to create a computerized database. The raw data of 44 full-text articles were strictly screened and converted to standardized units per parameter, e.g., g/100 g fatty acid methyl esters (FAMEs) and g/kg dry matter (DM), for all FA and tannin levels, respectively. However, 6 articles were excluded because of unbalanced or unclear data. In the present meta-analysis, the tannin criteria was also established on the basis of a prior meta-analysis [24]; in brief, the condensed and hydrolysable tannins in the included studies originated from plants and had been extracted. However, a comparison of each tannin type, for instance, condensed vs. hydrolysable tannins in a single treatment, was not provided. Comparisons of these results were provided in grouping studies. The comprehensive database consisted of 3712 dietary treatments in 38 publications, as shown in Table 1 (11 *in vitro* experiments/2098 treatments), Table 2 (14 *in vivo* experiments for CLAs in milk/580 treatments) and Table 3 (13 *in vivo* experiments for CLAs in meat/3712 treatments). There was an article that included both *in vitro* and *in vivo* experiments on CLAs in milk [27] and an article that compared CLAs in milk and meat *in vivo* [28]. Sensitivity and risk of bias analyses were performed for the selected publications. The risk of bias was based on sufficient data, the study type and the probability of confounding. Effect sizes were subjected to a meta-analysis model based on standardized mean differences (SMDs). Funnel plots, Egger’s tests for funnel plot asymmetry and fail-safe number calculations were used to assess publication bias, with *P <* 0.05 indicating significant publication bias [29] (see S2 Table for further information).

**Table 1:** Data tabulation of *in vitro* experiments.

No exp.	Reference	*In vitro* method	Inocula donor	Basal feed	Tannins source	Tannin level (g/kg DM)	Gas sampling (h)
1	Carreño, et al [30]	BCI	Sheep (Ewe)	TMR, Forage: Concentrate (50:50)	Chestnut (HT), Oak (HT), Quebracho (CT), Grape seed (CT)	20–80	24
2	Costa, et al [31]	GBI	Sheep	Dehydrated Alfalfa, concentrate and sunflower oil	Chestnut (HT), Quebracho (CT), Grape seed (CT), *Cistus ladanifer* (CT)	100	6
3	Costa, et al [22]	RBP	Sheep	Grass hay, concentrate, vegetable oil	Mimosa (CT), Chestnut (HT), Mimosa plus chestnut (MIX)	100	0
4	Guerreiro, et al [32]	HGT	Sheep (Merino Branco ram)	Maize, concentrate and sunflower oil	*Cistus ladanifer* (CT)	100	6
5	Ishlak, et al [33]	BCI	Cow (Holstein)	Forage: Concentrate (55:45)	Quebracho (CT)	100	24
6	Jayanegara, et al [34]	HGT	Cow (Brown Swiss)	Clover-ryegrass hay and concentrate	*Poa alpina* (HT), *Achillea millefolium* (HT), *Alchemilla xanthochlora* (HT), *Capsella bursapastoris* (HT), *Carum carvi* (HT), *Chrysanthemum adustum* (HT), *Crepis aurea* (HT), *Plantago atrata* (HT), *Rhinanthus alectorolophus* (HT), *Rumex arifolius* (HT), *Anthyllis vulnenaria* (HT), *Hedysarum hedysaroides* (HT), *Trifolium badium* (HT), *Castanea sativa* (HT), *Fraxinus excelsior* (HT), *Sambucus nigra (flowers)* (HT).	1–78	24
7	Jayanegara, et al [21]	HGT	Cow (Brown Swiss)	Hay (white clover), ryegrass and concentrate	*Acacia mangium*, *Acacia villosa*, *Albizia falcataria*, *Artocarpus heterophyllu*, *Calliandra calothyrsus*, *Canna indica*, *Carica papaya*, *Clidemia hirta*, *Cycas rumphii*, *Erythrina orientalis*, *Eugenia aquea*, *Hibiscus tiliaceus*, *Ipomoea batatas*, *Lantana camara*, *Leucaena diversifolia*, *Leucaena leucocephala*, *Manihot esculenta*, *Melia azedarach*, *Mimosa invisa*, *Morinda citrifolia*, *Myristica fragrans*, *Paspalum dilatatum*, *Persea Americana*, *Pithecellobium jiringa*, *Psidium guajava*, *Sesbania grandiflora*, *Swietenia mahagoni*.	2–220	24
8	Minieri, et al [35]	HGT	Sheep (Ewe)	Forage and concentrate	Quebracho (CT)	49	18
9	Szczechowiak, et al [27]	Bag incubate of RUSITEC	Cow (Polish Holstein-Friesian)	TMR, silage and concentrate	*Vaccinium vitis idaea* (CT)	4.5	24
10	Toral, et al [36]	BCI	Sheep (Ewe)	Hay (Alfalfa)	*Onobrychis viciifolia* (CT)	5–35	24
11	Vasta, et al [37]	GBI	Cow (Holstein-Friesian)	Hay and Hay plus	*Ceratonia siliqua* (CT), *Acacia cyanophylla* (CT), *Schinopsis lorentzii* (CT)	0.06–0.01	12

GBI, glass bottle incubation; BCI, batch culture incubation; HGT, Hohenheim gas test; RBP, rumen bacteria pellets; PMR, partial mixed ration; TMR, total mixed ration; DMI, dry matter intake; CT, condensed tannins; HT, hydrolysable tannins; DM, dry matter.

**Table 2:** Data tabulation of *in vivo* milk experiments.

No Exp.	Reference	Species	Basal feed	Tannins source	Tannin level (g/kg DM)	Adaptation period/long treatment (day)	Milking (time/day)
12	Alipanahi, et al [38]	Goat (Kurdish)	TMR, Alfalfa hay and concentrate	Oak acorn	9.1	21/42	2
13	Buccioni, et al [39]	Sheep (Comisana ewe)	Grass hay, rolled barley and concentrate	Chestnut (HT) and Quebracho (CT)	456–750	15/30	2
14	Buccioni, et al [40]	Sheep (Sarda ewe)	ryegrass (Lolium multiflorum), oat (Avena sativa) and white clover (Trifolium repens) (1:1:1) with grazing	Chestnut (HT)	80	90/210	2
15	Cabiddu, et al [41]	Sheep (Sarda ewe)	Pasture sulla with grazing	*Flowering sulla (Hedysarum coronarium L*.*)* (CT)	25–27.4	NS/30	2
16	de Lucena, et al [42]	Goat (Saanen)	Pornunca silage and concentrate (60:40)	Pornunca silage-based diets	11–48	20/80	2
17	Dschaak, et al [43]	Cow (Holstein)	TMR, Forage and concentrate (59:41)	Condensed tannin extract	30	14/21	2
18	Girard, et al [44]	Cow (Holstein)	TMR, a mixture of grass hay (86:10:4 of grass, legumes, and other species, respectively)	Sainfoin (CT), BirdSfooT trefoil bull (CT), BirdSfooT trefoil polom. (CT) in pellet forms	30.4–190.9	21/52	2
19	Henke, et al [45]	Cow (Holstein)	TMR, a mixture of grass silage, maize silage and concentrate (34:32:34)	Quebracho tannin extract (CT)	15–30	13/42	2
20	Kälber, et al [46]	Cow (Brown Swiss)	TMR, a mixture of grass silage, maize silage and ryegrass hay (56:26:18)	Buckwheat, phacelia, chicory	4.19–14.91	10/21	2
21	Lobón, et al [28]	Sheep (Churra Tensina ewe)	Permanent dam and pasture (composed of 68% grass, 20% leguminous plants, and 12% other species)	Quebracho (CT)	2	NS/NS	Weekly
22	Maamouri, et al [47]	Sheep (Sicilo-Sarde ewe)	Triticale pasture	*Acacia cyanophylla*	32.7	45/70	2
24	Miri, et al [48]	Goat (crossbred: Alpine × Beetal)	Hay and concentrate	Cumin extract	1–2	21/25	2
25	Szczechowiak, et al [27]	Cow (Polish Holstein-Friesian)	Mix silage and concentrate	*Vaccinium vitis idaea* (CT)	32.8	21/26	2
26	Toral, et al [49]	Sheep (Assaf ewe)	TMR, forage and concentrate (40:60)	Commercial tannin	8.7	14/30	2

PMR, partial mixed ration; TMR, total mixed ration; DMI, dry matter intake; CT, condensed tannins; HT, hydrolysable tannins; DM, dry matter; NS = not specific/mentioned.

**Table 3:** Data tabulation of *in vivo* meat experiments.

No Exp.	Reference	Species	Basal feed	Tannins source	Tannin level (g/kg DM)	Adaptation period/long treatment (day)	Slaughtered period (day of age)
27	Gesteira, et al [50]	Bulls (uncastrated Nellora)	TMR, forage and concentrate (40:60)	*Acacia mearnsii* (CT)	10–50	15/105	615 (16 h fasting)
28	Kamel, et al [51]	Sheep (Naomi lamb)	Alfalfa hay and concentrate	Commercial quebracho (CT)	20–40	15/70	160 (direct)
29	Lobón, et al [28]	Sheep (Churra Tensina lamb)	Permanent dam and pasture (composed of 68% grass, 20% leguminous plants, and 12% other species)	Quebracho (CT)	2	NS/35	reached 10–12 kg of BW
30	Luzardo, et al [52]	Sheep (Texel and Australian Merino crossbreed lamb)	Pasture, intensive grazing	*Lotus uliginosus* cv. E-Tanin (E-Tanin) and *Trifolium repens* cv. Zapicán (white clover, WC)	2.1–5.8	14/28	748 (direct)
31	Marume, et al [53]	Goat (Xhosa lop-eare)	Pasture, intensive grazing	*Acacia Karoo* (CT)	82.5	30/90	210 (direct)
32	Rana, et al [54]	Goat (crossbred (Alpine×Beetal)	Maize and concentrate	*Terminela chebula*	1.2–10.7	NS/90	270 (direct)
33	Sharifi, et al [55]	Sheep (Baluchi lamb)	TMR, forage and concentrate (63:37)	Grape seed (CT)	0.063–0.073	42/56	252
34	Staerfl, et al [56]	Bull (Brown Swiss×Limousin crossbred)	Maize silage and concentrate	*Acacia mearnsii* (CT)	141	24/280	reached 525 kg of BW
35	Vasta, et al [57]	Sheep (Comisana lamb)	Herbage and concentrate	Quebracho (CT)	40.4–40.6	7/60	105
36	Vasta, et al [58]	Sheep (Comisana lamb)	Alfalfa hay and concentrate	Carob pulp (CT)	27	7/60	105
37	Vasta, et al [12]	Sheep (Comisana lamb)	Herbage and concentrate	Quebracho (CT)	40.3	NS/60	105
38	Vasta, et al [59]	Sheep (Comisana lamb)	Alfalfa hay and concentrate	Quebracho (CT)	6.45	7/77	122
39	Willems, et al [60]	Sheep (Engadine and Valaisian Black Nose ram)	Ryegrass-clover pasture, intensive grazing	Grass, legume and herb (native pasture compound) in vegetative stage	0.30–1.64	30/93	277

PMR, partial mixed ration; TMR, total mixed ration; DMI, dry matter intake; CT, condensed tannins; HT, hydrolysable tannins; DM, dry matter; NS = not specific/mentioned.

### Statistical analysis

The analysis of the assembled data was performed with a statistical univariate meta-analysis approach [24, 61, 62], using the MIXED procedure of SAS version 9.4 [63]. The following model was applied:

$${\text{Y}}_{\text{ij}}={\text{P}}_{0}+{\text{P}}_{1}{\text{Q}}_{\text{ij}}+{\text{R}}_{\text{i}}+{\text{p}}_{\text{i}}{\text{Q}}_{\text{ij}}+{\text{e}}_{\text{ij}}$$ (1)

where Y_ij_ = the dependent variable, P_0_ = the overall intercept across all experiments (fixed effect), P_1_ = the linear regression coefficient of Y on X (fixed effect), Q_ij_ = the value of the continuous predictor variable (supplementary tannin level), R_i_ = the random effect of experiment _i_, pi = the random effect of experiment _i_ on the regression coefficient of Y on X in experiment _i_, and e_ij_ = the deniable residual error. To input the CLASS statement, the variable “REFERENCENO” was applied without any quantitative information. Additionally, data were calculated according to the number of animal replicates in each experiment [63] and scaled to 1 to avoid misconception due to unequal variances among the experiments. In the fixed-effect model, small studies received lower weightings and higher weights were applied to larger studies (based on the number of measurements).

Outliers were identified by examining a mixed model with a maximum likelihood (ML) approach. For example, the model included the METHOD = ML; COVTEST; and PARMS statements followed by the EQCONS = 2 option. An unstructured variance-covariance matrix (type = un) was confirmed as the random part of the model. The significant differences among intercept, slope and regression coefficients were accepted.

Additionally, CLA amounts in milk and meat sources could not be compared directly in each study. It was possible to compare the total CLA data, including *in vitro* and *in vivo* observations, thoroughly. Because of incomplete data regarding the included variables, the present meta-analyses were technically performed based on the available data of individual variables.

## Results

### Search results, sensitivity and bias assessments

**Fig 2:**
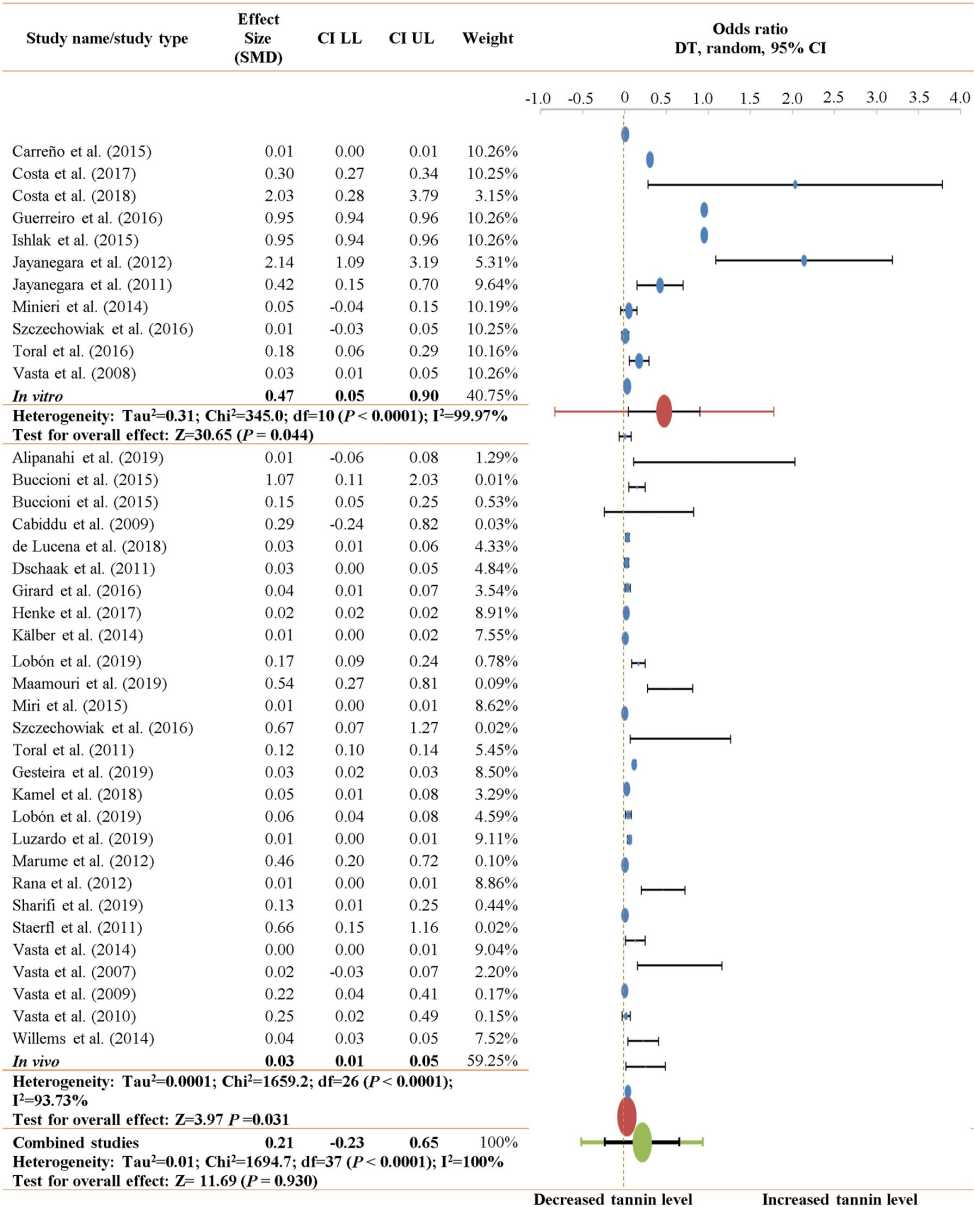
Forest plots for the links between supplementary tannin levels and CLA.

**Fig 3:**
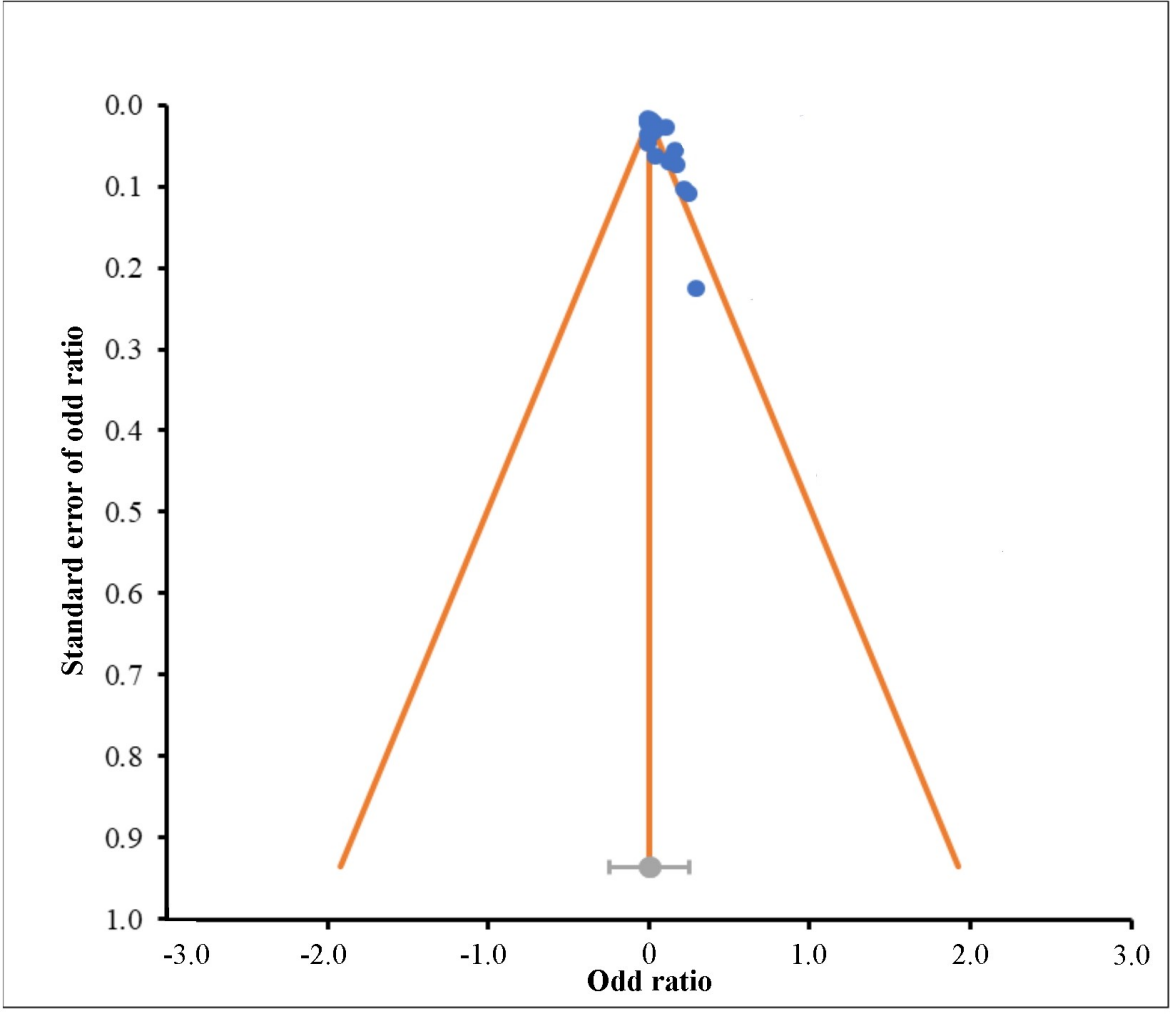
Funnel plots for the links between supplementary tannin levels and CLA.

As depicted in Fig 1, the present meta-analysis was derived from a strict dataset. There was a 99.99% rejection rate during the collection of publications, which exists in academic settings around the world, especially in the USA, P.R. China, Germany, Japan and India (S1 Fig). A possible reason for this result was the inconsistent parameters of CLA synthesis and the types of observations. Regarding the selected articles (Tables 1–3), sensitivity is depicted in a forest plot (Fig 2), and risk of bias is presented in a funnel plot; the risk of bias for the CLA data in all the types of studies in the present meta-analysis was not significant (*P* = 0.067) (Fig 3). The sensitivity and risk of bias for the other parameters is provided in the supplementary data.

### The effective level of tannins

The results of the meta-analysis of the regression lines for predicting the outcomes of *in vitro* batch culture experiments in terms of dietary tannins as a predictive variable are presented in Table 4. Cis-9, trans-11, 18:2 CLA increased (*P* < 0.0001 in linear equation) with increasing tannin supply, with an R^2^ of 0.6422. Likewise, VA increased (*P* < 0.05) with increasing tannin supply, with an R^2^ of 0.6242. However, SA decreased (*P* < 0.001) with increasing tannin supply, with an R^2^ of 0.6701. These results supported the change in the apparent FA proportions expressed as g/100 g FAMEs, and PUFAs were increased (*P* < 0.001) after tannins were supplemented (R^2^ = 0.5020). Dietary tannins were not associated with monounsaturated fatty acid (MUFA) and SFA proportions. In addition, total gas production decreased with increasing (*P* < 0.0001) tannin supply, with an R^2^ of 0.1736, but dietary tannins did not change the rumen fermentation characteristics, including total volatile fatty acids (VFAs, mmol/L) and their acid components. According to the statistical meta-analysis results, the range of tannin levels to meet the aforementioned outcomes in the *in vitro* studies was approximately 0.1–20 g/kg DM (*P* < 0.001).

**Table 4:** The predicted equation of *in vitro* batch culture experiments^a^.

Response parameter	N	Parameter estimation							
		Intercept	SE intercept	P intercept	Slope	SE slope	P slope	RMSE	R^2^
FA supplementation (g/100 g FAME)									
C18:3 n-3	2098	0.1161	0.0081	0.0889	0.0349	0.0024	0.0963	17.3120	0.0891
C18:2 n-6	2098	0.0973	0.0068	0.0600	0.0012	0.0003	0.0780	14.3330	0.0899
C18:1 n-9	1858	0.1537	0.0074	0.0773	0.0034	0.0012	0.0611	15.4080	0.1870
Gas production (mL/g OM)	317	2.0662	0.2540	0.0005	-0.0005	0.0001	<0.0001	125.0300	0.1736
Total VFA (mmol/L)	1392	0.5722	0.0216	<0.0001	0.0011	0.0001	0.0622	23.1320	0.3349
C_2_	1392	0.0156	0.0081	0.1198	0.0016	0.0008	0.0938	8.6198	0.2700
C_3_	1392	0.0058	0.0044	0.3412	0.0065	0.0011	0.0521	7.3570	0.1200
C_4_	1392	0.0024	0.0036	0.8638	-0.0100	0.0046	0.0790	3.8071	0.0300
C_5_	1152	0.0301	0.0051	0.0018	0.0071	0.0052	0.2982	4.0477	0.3570
Iso-C_4_+Iso-C_5_	576	0.1648	0.0064	<0.0001	5.4761	5.5837	0.6116	0.1791	0.5340
FA profile (g/100 g FAME)									
Cis-9, trans-11, 18:2 (CLA)	2098	0.0044	0.0006	0.0008	0.2009	0.0146	<0.0001	1.3041	0.6422
Trans-11 18:1	2098	0.0077	0.0021	<0.0001	0.0392	0.0036	0.0228	4.5276	0.6242
C18:0	2098	0.0337	0.0056	0.0016	-0.0001	0.0015	0.0017	11.9020	0.6701
SFA	1842	0.0279	0.0121	0.0699	0.0008	0.0019	0.9898	22.4760	0.2973
MUFA	1842	0.0205	0.0108	0.1294	-0.0018	0.0021	0.7173	20.0980	0.1956
PUFA	1842	0.0025	0.0026	0.6038	0.0458	0.0048	0.0002	4.8936	0.5020

C_2_, acetate; C_3_, propionate; C_4_, butyrate; C_5_, valerate; VFA, volatile fatty acid; FA, fatty acid; FAME, fatty acid methyl esters; CLA, conjugated linoleic acid; SFA, saturated fatty acid; MUFA, mono-unsaturated fatty acid; PUFA, poly-unsaturated fatty acid; DM, dry matter; OM, organic matter; N, total data used; SE, standard error; P, *p* value; RMSE, residual mean square error; R^2^, coefficient of determination.

^a^Outcomes are averages deriving from tabulated data in Table 1 calculated by proc mixed.

Furthermore, the results of the meta-analysis of the regression lines to predict the outcomes of *in vivo* experiments in terms of dietary tannins as a predictive variable are presented in Table 5. The apparent CLA proportions in both milk and meat increased with increasing tannin supply (*P* < 0.0001 and *P* < 0.001, with R^2^ values of 0.8352 and 0.6711, respectively). However, these results indicated that SA decreased with increasing tannin supply (*P* < 0.01). Additionally, there was a significant, though minor, increase in VA (*P* < 0.05) with increasing tannin supply, although the relationship was nonsignificant (R^2^ < 0.6). These results in milk were similar to those in apparent FA proportions in meat, expressed in g/100 g FAMEs. Interestingly, Δ^9^ desaturation and the CLA index, whether in milk or meat, increased with increasing tannin supplementation (*P* < 0.05 and *P* < 0.001, with average R^2^ values of 0.2318 and 0.4382, respectively). Similar to the *in vitro* outcomes, dietary tannins did not change the rumen fermentation characteristics, including total VFAs (mmol/L) and their acid components. Therefore, the range of tannin levels necessary to obtain the abovementioned results of the *in vivo* studies was approximately 2.1–80 g/kg DM (*P* < 0.001).

**Table 5:** The predicting equation of *in vivo* batch culture experiments^a^.

Response parameter	N	Parameter estimation							
		Intercept	SE intercept	P intercept	Slope	SE slope	P slope	RMSE	R^2^
FA supplementation (g/100 g FAME)									
C18:3 n-3	1284	0.0004	0.0003	15.8459	-0.0022	0.0423	0.0453	34.9540	0.0100
C18:2 n-6	1342	0.0595	0.0065	<0.0001	-0.0002	0.0001	0.0528	15.2890	0.0581
C18:1 n-9	1342	0.0125	0.0029	<0.0001	0.0160	0.0010	0.0600	6.8641	0.0133
Total VFA (mmol/L)	117	0.0429	0.0126	0.0182	0.0026	0.0013	0.2952	25.1710	0.0921
C_2_	117	0.0347	0.0050	0.0010	0.0001	0.0072	0.5440	9.9188	0.2988
C_3_	117	0.0006	0.0084	0.0025	0.0324	0.0043	0.0694	16.7870	0.0000
C_4_	117	0.0008	0.0026	0.0098	-0.0003	0.0019	0.4956	5.1379	0.0006
C_5_	73	0.0031	0.0005	0.0004	-0.0014	0.0074	0.9931	0.8895	0.3975
Iso-C_4_ + Iso-C_5_	31	0.0003	0.0006	0.9454	-0.0009	0.0144	0.8202	1.0553	0.0081
FA profile in milk (g/100 g FAME)									
Cis-9, trans-11 18:2 (CLA)	580	0.0159	0.0011	<0.0001	0.0303	0.0033	<0.0001	2.2603	0.8352
Trans-11 18:1	580	0.0006	0.0026	0.0188	0.0034	0.0014	0.0442	5.5867	0.4001
C18:0	580	0.0015	0.0016	0.0622	-0.0102	0.0026	0.0024	3.5275	0.5100
SFA	580	0.0044	0.0017	0.0363	-0.0131	0.0036	0.0157	3.7550	0.1070
MUFA	580	0.0017	0.0022	0.7853	-0.0037	0.0016	0.0856	4.6519	0.0010
PUFA	580	0.0036	0.0013	0.0214	0.0146	0.0024	0.0017	2.6944	0.3891
Desaturation index	580	0.00003	0.00002	0.2471	0.0239	0.0025	0.0002	0.0456	0.3500
CLA index	580	0.0022	0.0041	0.9667	0.0088	0.0012	0.0004	8.8529	0.5441
FA profile in longissimus dorsi muscle (g/100 g FAME)									
Cis-9, trans-11, 18:2 (CLA)	1034	0.0083	0.0007	<0.0001	0.0059	0.0018	0.0002	0.4590	0.6711
Trans-11, 18:1	1034	0.0012	0.0017	0.8516	0.0072	0.0023	0.0107	1.1123	0.5350
C18:0	1034	0.1145	0.0090	<0.0001	-0.0057	0.0008	0.0001	5.8240	0.6131
SFA	1034	0.1386	0.0079	<0.0001	0.0144	0.0009	0.0653	5.0920	0.2321
MUFA	1034	0.0165	0.0092	0.0031	0.0149	0.0020	0.0001	5.9406	0.3152
PUFA	1034	0.0803	0.0115	0.0002	-0.0227	0.0009	0.2882	7.4543	0.4526
Desaturation index	1034	0.0008	0.0001	0.0031	0.0238	0.0091	0.0251	0.0454	0.1137
CLA index	1034	0.2252	0.0118	<0.0001	0.0122	0.0025	0.0009	7.2738	0.3324

C_2_, acetate; C_3_, propionate; C_4_, butyrate; C_5_, valerate; VFA, volatile fatty acid; FA, fatty acid; FAME, fatty acid methyl esters; CLA, conjugated linoleic acid; SFA, saturated fatty acid; MUFA, mono-unsaturated fatty acid; PUFA, poly-unsaturated fatty acid; DM, dry matter; OM, organic matter; N, total data used; SE, standard error; P, p-value; RMSE, residual mean square error; R^2^, coefficient of determination. Desaturation and CLA indices were calculated as given by Corl, et al [64] and Schennink, et al [65] reports, respectively.

^a^Outcomes are averages deriving from tabulated data in Tables 2 and 3 calculated by proc mixed.

### The regression method

**Fig 4:**
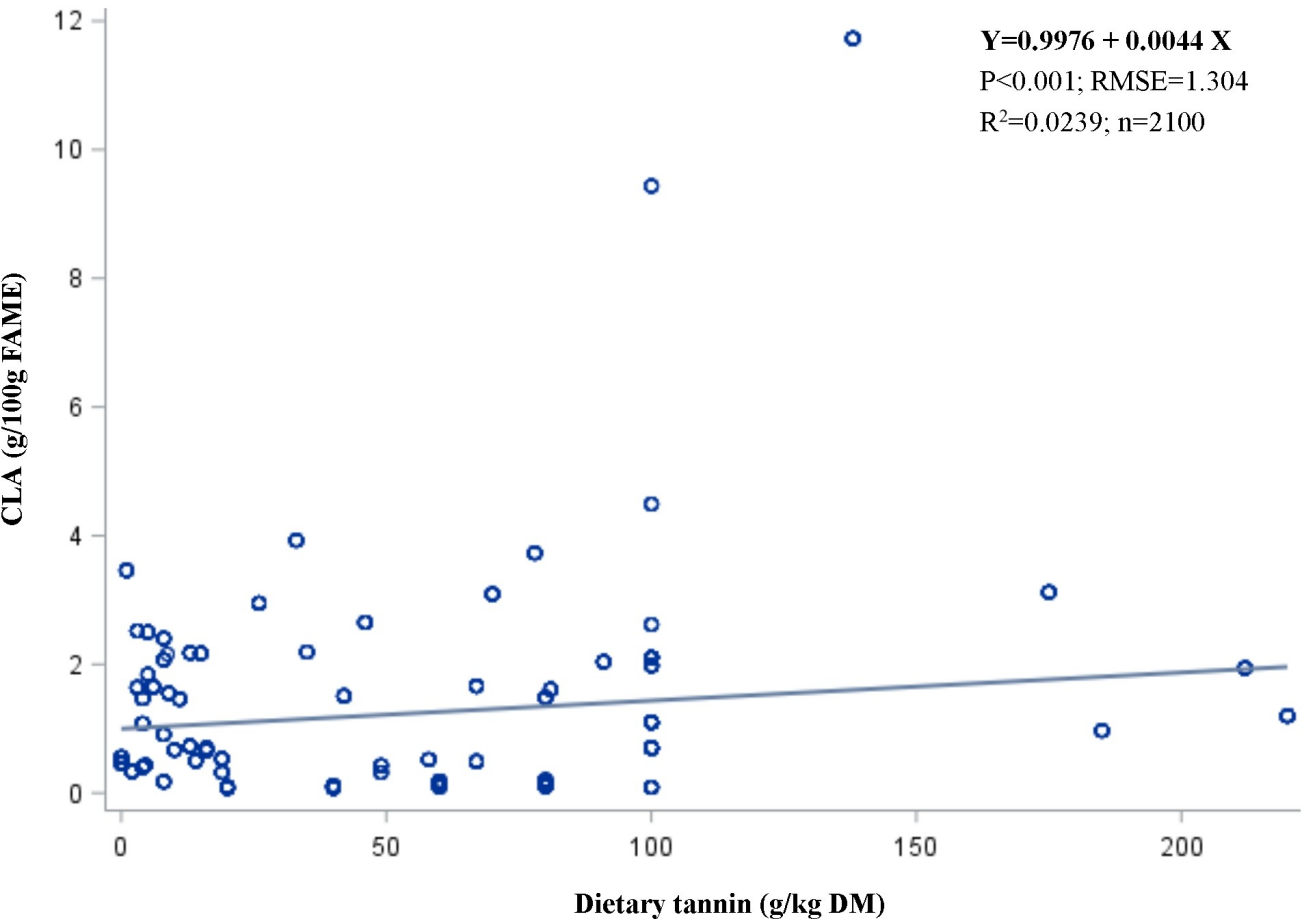
The regression relationships between the *in vitro* and *in vivo* CLA deposition in milk.

**Fig 5:**
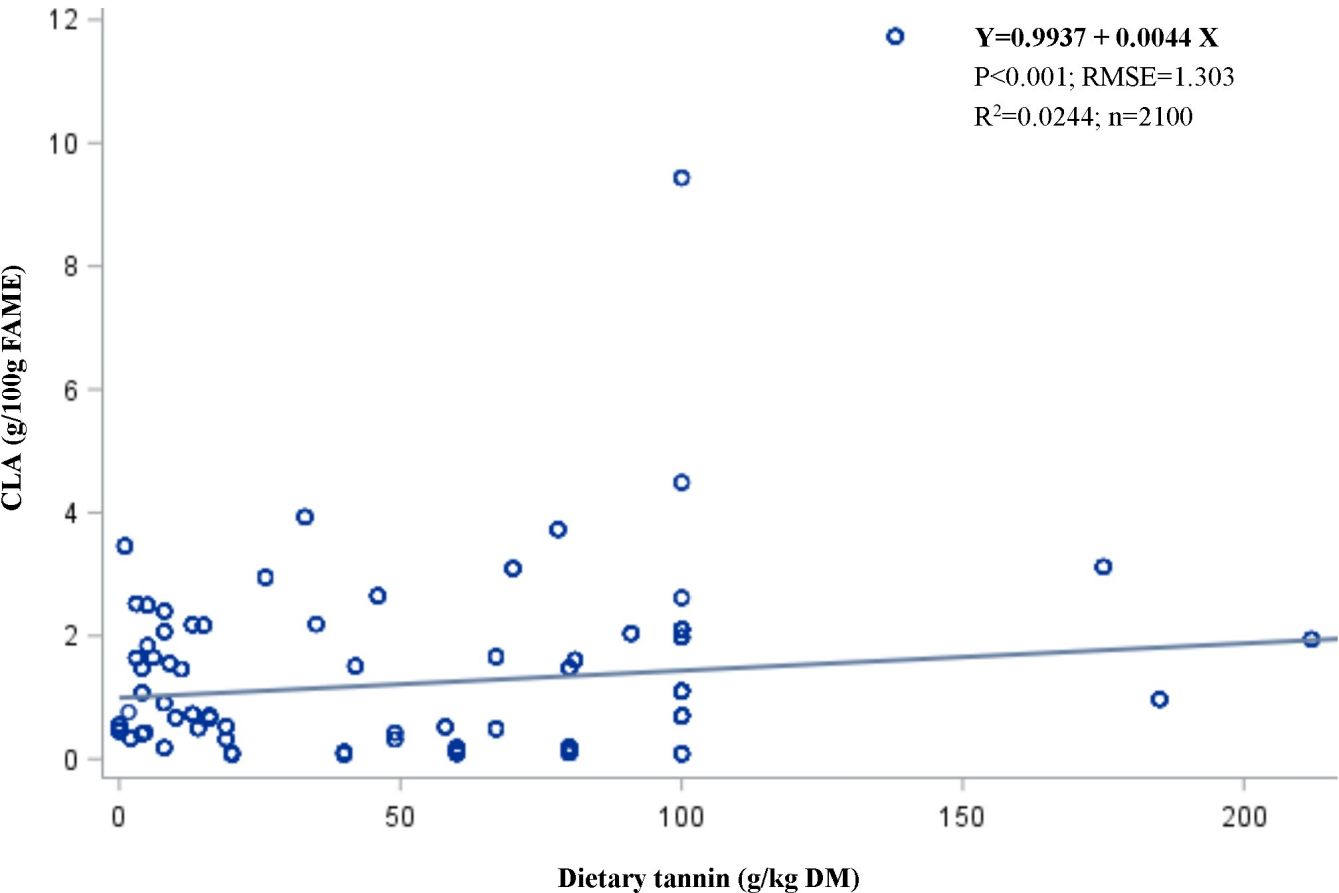
The regression relationships between the *in vitro* and *in vivo* CLA deposition in meat.

The regression results between the *in vitro* and *in vivo* CLA deposition in milk are depicted in Fig 4, and the results between the *in vitro* and *in vivo* CLA deposition in meat are shown in Fig 5. These relationships were expressed linearly rather than quadratically. Clearly, there were no relationships among them (R^2^ < 0.1).

## Discussion

Regardless of previous publications, including meta-analyses, in which methane mitigation by dietary tannins in ruminant feeding regimens was critical, there are, to our knowledge, no investigations on altered biohydrogenation improvements in CLAs due to dietary tannin supplementation supported by numerical data collection. A prior meta-analysis regarding dietary FAs and feeding regimens found variable C18 FA amounts in milk, revealing an inextricable link with different types of feeding regimens [23]. Consistent with other summaries, C18 FA concentrations in meat were modified through feeding approaches, such as supplementation with dietary C18 FAs [8]. The C18 FA profiles had curvilinear relationships with CLAs, which were synthesized in an extremely limited range of 0.34–1.07% in total fat in milk and 0.12–0.68% in total fat in meat. These studies considered multiple ruminant diets; breeds; ages; antinutritional feed additives, such as ionophores; and synthetic mixtures of dietary CLAs. The results of the present meta-analysis of data on FA supplementation regimes, breeds, ages and applied techniques did not detect a bias, indicating the sole role of tannins in CLA deposition. As expected, the present results accurately predicted the suitable level of tannins *in vitro* and *in vivo* and thus confirmed the results under *in vitro* to *in vivo* conditions.

### The types of dietary tannins as regulators of ruminal biohydrogenation

The results of the present meta-analyses showed that dietary tannins in ruminant diets modulated CLA synthesis, inhibiting ruminal biohydrogenation. From a chemical standpoint, tannins are polyphenolic biomolecules that are roughly defined by broadly diverse oligomers and polymers. Frutos, et al [66] reviewed applied tannin classifications with regard to ruminant benefits. Before an in-depth understanding of this potent secondary metabolite was reached, tannins had been commonly separated into two groups. Hydrolysable tannins (HTs), or pyrogallol-type tannins, are created from carbohydrate cores and hydroxyl groups. Gallic and ellagic acids are examples of these tannin groups, and sometimes these tannins are bonded to other hydroxyl compounds such as flavonoids [67]. The second group is condensed tannins (CTs), which can be defined as nonbranched polymers with poor carbohydrate contents; this group has a higher molecular weight than HTs [68]. There is also another group of tannins characterized as catechins (i.e., tannins present in green tea leaves). Similar to condensed tannins, they also have a characteristic carbon skeleton in their structure without sugar residues [69]. A mixed tannins (MTs) (composed of HT and CT compounds) may occur in some plants.

Over the past 3 decades, CTs, HTs and MTs have been applied to ruminant diets *in vitro* and *in vivo* as regulators of ruminal biohydrogenation. Chestnut (HT), oak (HT), quebracho (CT), and grape seed (CT) at 20, 40, 60 and 80 g/kg, respectively, increased the PUFA content of FAs in the rumen content after 24 hours of incubation, whereas doses of 80 g/kg tended to promote the accumulation of cis-9, trans-11 CLA [30]. An investigation involving a shorter incubation time of 6 h by Costa, et al [31] found that grape seed (CT) increased the disappearance of C18 FA in the diet, resulting in the increased production of cis-9, trans-11 CLA and VA, with a remaining amount of SA. It seemed likely that CTs had the ability to control the production of PUFAs and MUFAs during ruminal biohydrogenation, and the CLA transformation from dietary unsaturated fatty acids (UFAs) in the diet occurred rapidly. However, Jayanegara, et al [34] monitored 18 species of alpine forage plant for the disappearance and appearance of C18 profiles, and CT and HT concentrations were compared. HTs but not CTs showed a clear positive correlation with the appearance of cis-9, trans-11 CLA. Jayanegara, et al [21] also conducted a systematic investigation of the effects of 27 tropical forages (mainly tree and shrub leaves) on the extent of biohydrogenation. The MT contents of tropical forages had positive correlations with the production of cis-9, trans-11 CLA (R^2^ = 0.27) and negative correlations with the production of SA (R^2^ = 0.18), consistent with other reports that observed alternations in milk and meat [28, 49, 54, 60, 70].

Despite the increase in cis-9, trans-11 CLA and decrease in SA, dietary CTs, HTs and MTs had a slight impact on total VFA and ruminal microorganisms [22, 27, 36]. Decreasing concentrations of VFA and *Butyrivibrio proteoclasticus* (SA producer) occurred with increasing cis-9, trans-11 CLA due to supplementation with CT and HT at doses of 100 g/kg DM. CTs had greater efficiency in reducing SA producers than HTs [22]. However, these supplements retained cis-9, trans-11 CLA. In contrast, the same dose of CTs derived from *Cistus ladanifer* extract did not change the total VFAs and increased degradation of available PUFAs in the diet due to the isomerization of cis-9, trans-11 CLA [32]. Further, a relatively low dose of other CT sources, such as *Vaccinium vitis-idaea* dosed at 4.5 g/kg DM, was confirmed to reduce SA producers, demonstrated by the declining relative DNA abundance of *Butyrivibrio proteoclasticus* [27]. In addition, other tannin levels were tested by Vasta, et al [37] using CT sources from carob (*Ceratonia siliqua*), Acacia leaves (*Acacia cyanophylla*) and quebracho (*Schinopsis lorentzii*) at doses of 0.06–0.1 g/kg DM for their effects on ruminal biohydrogenation *in vitro*. Tannins increased VA by 23% and decreased SA by 16% of the total FAs without altering the total VFAs, which was consistent with other observations using oak (HT) at a dose of 20 g/kg DM [30].

Thus, the types of dietary tannins seemed to have identical effects on ruminal biohydrogenation. Considering a sufficient FA content in the diet, bioactive tannins exhibited promising antimicrobial properties in many studies and could be inhibitors of lipogenic processes, resulting in increased production of cis-9, trans-11 CLA and VA in ruminant-derived products. However, the doses of dietary tannins should be reconsidered regard of unexpected secondary metabolite behavior of FA synthesis in relation to other aspects of the rumen.

### The effects of dietary tannins on CLA biosynthesis

CLAs are natural UFAs and are defined as primary intermediate products generated from their shortened conjugated precursors (LA and ALA) by lipolysis, isomerization and biohydrogenation by rumen lipid microorganisms. During biohydrogenation, CLAs in the cis-9, trans-11 18:2 form and their derivatives are isomerized rapidly [2, 9]; subsequently, the remaining substrate terminates biohydrogenation. Primary CLA production occurs by desaturation by removing two hydrogen atoms from trans-11 18:1 to form cis-9, trans-11 18:2 with the Δ^9^-desaturase enzyme [8, 12, 13]. In other words, the method of synthesizing CLAs involves altering biohydrogenation pathways by modifying CLA precursors in the diet [8].

Except for CLA precursors such as LA and ALA, low amount of dietary UFA in a form of cis-9 C18:1 (OA: oleic acid) can be also found in forages, cereals and oils. Additionally, if the feeding regimen uses a grazing system, whether the natural polyphenols in the diet contain tannins should be reconsidered [71]. The present meta-analysis, which did not detect a bias, revealed that dietary tannins in animal diets had linear relationships with increased concentrations of cis-9, trans-11 CLA and VA in milk and meat. Forages that produce tannins could be considered additional organic matter for animal diets rich in FAs. This finding was reliable, as the results were in accordance with those of a previous meta-analysis by Średnicka-Tober, et al [25] who observed cis-9, trans-11 CLA and VA in excessive concentrations in milk from animals maintained on organic farms that consumed more forage than those on conventional farms, consistent with other studies [23]. Notably, other factors, such as plant wilting and leaf breakage during hay production (and to some extent prior to ensiling), in conventional farming systems may be important, as decreasing FA concentrations in forage may result from oxidative loss [72]. However, natural fresh tannins and other secondary metabolites of pasture plants in organic grazing systems are inevitably consumed by animals, resulting in optimum FA intake, which seems to encourage subsequent biohydrogenation [21].

Lourenço, et al [2] summarized the role of rumen microbial lipids in practical biohydrogenation. When CLAs were synthesized in the first biohydrogenation, group A of bacteria (*Butyrivibrio fibrisolvens*) was shown to produce CLAs from C18 FA with the enzyme linoleate isomerase, whereas lipolysis, isomerization and biohydrogenation were undertaken by group B of bacteria (*Butyrivibrio proteoclasticus*). Eighteen Comisana ewes at 122 ± 6 d in milk were provided quebracho (CT) and chestnut (HT) at doses of 52.8 g/kg DM, and the presence of *Butyrivibrio fibrisolvens* and *Butyrivibrio proteoclasticus* in the rumen liquor was observed and compared [70]. The presence of *Butyrivibrio fibrisolvens* increased (5-fold) in ewes fed the CT diet and (3-fold) in ewes fed HT diet, when it was compared with rumen liquor from ewes fed the control diet. The presence of *Butyrivibrio proteoclasticus* decreased (15-fold) in ewes fed the CT diet and (5-fold) in ewes fed the HT diet, similar to other results in different breed [13, 48]. However, the mechanisms by which tannins reduce *Butyrivibrio proteoclasticus* in different dietary components (CTs vs. HTs) are not entirely clear considering various basal substrates, tannin dose (type included), age, breed and environmental farming system [66, 73, 74]. In comparison, twelve multiparous Polish Holstein-Friesian cows (600 ± 30 kg body weight) in their 5-6th month of lactation were fed a basal diet with *Vaccinium vitis-idaea* extract at 4.5 g/kg DM, which unfortunately resulted in a nonsignificantly different relative abundance of *Butyrivibrio proteoclasticus* and *Butyrivibrio proteoclasticus* DNA.

Obviously, the relatively high amounts of CLAs and VA and relatively low amounts of SA in the present meta-analysis revealed that the secondary metabolites like tannins had a greater ability to suppress *Butyrivibrio proteoclasticus* than to suppress *Butyrivibrio fibrisolvens*. The putative reason for that fact is the difference in the semipermeable membrane in these bacteria. *Butyrivibrio fibrisolvens* likely has durable impermeable barrier [75]. As a result, the effect of dietary tannins was halted by the outer membrane of this gram-negative organism.

### The effects of dietary tannins on *de novo* synthesis and endogenous desaturation

Regarding CLAs in milk synthesis, after UFA incorporation into the diet of ruminants, two consecutive metabolic processes occur. First, triglycerides from the diet (LA 85%; ALA 93%, [11]) are degraded into free FAs, expressed as rumen-escaped PUFAs in the mammary gland (elucidated above), thereafter undergoing *de novo* FA synthesis and secreted as short- and medium-chain FAs in milk (Sc and Mc FAs). Lower CLA and VA contents were found in milk from Saanen goats fed Pornunca silage-based diets containing different levels of tannins (11, 28, 36 and 44 g/kg DM) [42]. It seems likely that the dietary tannins encouraged *de novo* FA synthesis to transform CLA and VA to Sc and Mc FAs. However, this was inconsistent with the results of Kälber [46] who supplemented diets with different vegetative and reproductive stages of buckwheat, chicory, and phacelia at doses of 4.19–14.91 g/kg DM, resulting in inhibited *de novo* FA synthesis. Hence, these results could indicate that dietary tannins influence *de novo* FA synthesis irregularly in the mammary gland depending on the density of tannins [66, 76].

In secondary metabolism, available free FAs are also metabolized and biohydrogenated to form the end-product of rumen biohydrogenation (mainly to C18:0) and isomers of MUFAs and PUFAs (*trans* and conjugated FAs). These intermediate products of rumen biohydrogenation are absorbed in the gut, transferred to the mammary gland by the bloodstream and finally secreted into milk fat, affecting the milk FA composition [11]. Despite being directly absorbed and secreted into milk, approximately one-half of FAs, especially Sc and Mc FAs, in the milk (molar percent) of dairy ruminants are acquired from *de novo* FA synthesis that occurs in the mammary gland [77, 78]. The intermediate products of rumen biohydrogenation are synthesized into simple SFAs (almost all of 4:0–14:0 and 50% of 16:0) in the mammary gland, and specific FAs of C10-C19 undergo Δ^9^ desaturation by stearoyl-CoA desaturase (SCD) in the endoplasmic reticulum of the mammary gland, resulting in palmitoleoyl-CoA, oleyl-CoA and the main isomers of CLAs, which represent cis-9, trans-11 18:2 and trans-7, cis-9, accounting for approximately 75–90% and 3–16% of the total CLAs, respectively [2, 9, 11]. Among the specific FAs of C10-C19, VA is reported to be a main substrate for SCD, producing an abundance of cis-9, trans-11 18:2 [79, 80]. As mentioned above, in the current study, dietary tannins did not regularly affect *de novo* FA synthesis, but it seemed likely that dietary tannins have a positive effect on lipolytic activity by regulating the conversion of VA to SA in mammary tissue [13, 48, 70]. In addition, other lipids of microbial, as well as protozoa and fungi origin, should be considered. Tannins have been extensively reported in previous studies [27, 81, 82], and decreasing the total amount of ruminal protozoa was the predominant effect.

Evidence of lipolytic activity in protozoa is strongly inconsistent [2]. Recent publications have shown that there is a positive relationship between the abundance of rumen ciliated protozoa and the proportion of cis-9, trans-11 CLA and VA; this relationship has been shown for holotrichs, entodiniomorphids, *Isotricha* and *Epidinium* [16]. Additionally, a linear relationship between decreasing SA and increasing amounts of total ciliates, holotrichs, entodiniomorphids and *Isotricha* has been shown [16]. In addition, rumen protozoa, especially *Epidinium* spp., were reported to account for approximately 30–40% of lipolysis activity by adhering to the plant surface (possibly in feedstuff) using an anterior pleated zone, resulting in the easy breakdown of cellular membranes [14]. Hence, rumen protozoa were confirmed to be a rich source of PUFAs, especially CLAs and VA, due to the ingestion of more chloroplasts [15], but these organisms did not induce Δ^9^ desaturation [15, 17] associated with bacteria lipids. In other words, obtained CLAs and VA by protozoa were not synthesized from available FAs and/or SA [2]. Additionally, considering only the contribution of protozoa in biohydrogenation with respect to the activity of ingested or associated bacteria, Lourenço, et al [2] suggested limiting protozoa because *Butyrivibrio fibrisolvens* had more important role in synthesizing CLAs, affecting ruminal biohydrogenation.

In term of CLAs in meat, its biosynthesis occurs through a similar process, and the influence of dietary tannins is comparable to that in CLAs in milk [12, 27, 28, 46, 49, 54]. That is, the different rumen microorganism lipids interfered with biohydrogenation enzymes, but FA transformation was essentially equal. The FA composition, especially the CLAs in ewe milk or suckling lamb meat, was observed in thirty-nine ewes and lambs offered 10% quebracho tannins in their total diets (2 g/kg DM) [28], and differences between pasture forage and hay in dams were included. The ewes and lambs that produced the target CLAs were compared and exhibited a 94 ± 10.4% similarity score. According to this result, Lobón, et al [28] suggested that the use of quebracho to improve biohydrogenation should be wisely excluded. In addition, *Acacia mearnsii* extract was investigated in serial doses at 10, 30 and 50 g/kg DM in total mixed rations (TMRs), forage and concentrate (40:60), and the concentrations of PUFA in meat were observed [50]. The desaturase index was reported to be increased after supplementation with dietary tannins, but the amount of CLAs was decreased. This result indicated that *Acacia mearnsii* extract (CT) affected the animal performance with regard to the concentration of PUFAs in milk and meat. Regarding tannins, the differences in the desaturation rate in dairy and beef cattle were associated with FA availability in diets and feeding regimens, as shown in previous studies [8, 11, 23].

Dietary tannins have been well known not only to suppress antimicrobial properties but also to indirectly influence Δ^9^ desaturase expression by regulating fat and protein absorption [12, 54, 83]. Nonetheless, regulating these metabolisms depends on FA substrate availability in the mammary gland and/or adipose tissue [27, 84]. Hence, this feature of biohydrogenation cannot be excluded. The present meta-analysis showed that desaturase and CLA indices increased when dietary tannins increased with decreasing SFA content. Notably, ruminant fat is more saturated than fat from monogastric animals. This result might suggest that the enzymes involved with SFA (i.e., acetyl-CoA carboxylase and FA synthase) have been activated by bioactive tannins, tending to have a greater performance of Δ^9^-desaturase. This suggestion is supported by studies [12, 54, 85] showing that a Δ^9^-desaturase activity is fairly responsive to the dietary tannins as interpreted by increased concentrations of MUFAs and PUFAs. Ultimately, there is a putative theory that bioactive tannins (CTs and HTs) with abundant FAs in the diet are regulators that indirectly influence the endogenous desaturation process via alterations in absorbed FA and protein. In other words, bioactive tannins seem to have multiple roles in modulating the endogenous processes by changing microorganisms and enzymes with abundant FA substrates and suitable doses of tannins. The present meta-analysis also confirmed that desaturation rates between dairy and beef animals were relatively comparable to dietary tannins.

### The susceptibility of the observed techniques to CLA biosynthesis

The results of *in vitro* observations were examined to confirm the utility of *in vivo* studies when the results are compared in the same unit. According to the present meta-analysis, the units for CLA and dietary tannins were consistently g/100 g FAMEs and g/kg DM, respectively. However, CLA production derived from *in vitro* studies represented only semiactual biohydrogenation. The diets containing FAs and tannins were isomerized by only rumen microorganisms from rumen donors in the first step of biohydrogenation. Nevertheless, the majority of CLA biosynthesis endogenously occurs during the desaturation stage and utilizes lipid enzymes from the digestive tract [8, 11, 23]. Compared to the respective controls, CLA concentrations from consecutive observations, namely, the rumen stimulation technique (RUSITEC) using cannulated cows and productive cows *in vivo*, showed inconsistent results [27]. This finding indicated that it was difficult to predict *in vivo* results considering *in vitro* CLA biosynthesis. In contrast, Lobón, et al [28] conducted *in vivo* studies on ewes and lambs and found comparable CLA production values. The *in vitro* approach provides a low-cost starting point to screen for an increase in CLA contents due to bioactive tannins; however, the provision of tannin regulators in diets enriched with FAs *in vivo* is strongly recommended.

## Conclusion

This meta-analysis, which included a large amount of data from valid publications and did not detect a bias, provides a prediction of suitable dietary tannins as extracts or plants that could be supplemented in rumen diets with a fit design to modulate the effect of CLA synthesis on biohydrogenation. The recommended doses of dietary tannins did not exceed 20 g/kg DM and 80 g/kg DM for *in vitro* and *in vivo* studies, respectively. However, the ratios of forage to concentrate were similar (nearly 50:50). *In vitro* studies without animals may be rapid, simple and low-cost approaches, but the results sometimes exhibit unexpected and questionable outcomes. Hence, the *in vivo* approach was more suitable for the direct observation of FA transformation. Further, the results suggest the critical need to identify or select the origin of tannins. If tannins are derived from commercial products/extraction, the purity of tannins should be strictly evaluated. Likewise, regarding tannins from plants, specific tannin-binding polymers and other hydroxyl groups should be widely considered. More research on other hydroxyl groups, such as flavonoids, is required to gain a better understanding of the extent of CLA synthesis (not only cis-9, trans-11 18:2 isomers but also trans-10, cis-12 18:2 isomers).

## Supporting information

S1 Table Full electronic search strategy on using keywords. (DOCX)

S2 Table Results of the standard meta-analysis, sensitivity analysis and risk of bias for other parameters relate to biohydrogenation. *n*, number of data points included in the comparison; SMD, standardized mean difference; CI LL, confidence interval lower; CI UL, confidence interval upper; *P value < 0.05 indicates significance of the difference among *in vitro* and *in vivo* observations; ^€^Heterogeneity and the I^2^ Statistic; ^δ^Ln ratio was calculated as Ln (*in vivo/in vitro* × 100%). ^§^*P* < 0.05 indicates significant publication bias.
(XLSX)

S1 Checklist PRISMA checklist. (DOC)

S1 Fig Top academic settings around the world involves meta-analysis database. The databases were searched from January 1992 to March 2019.
(TIF)
