# Supplementary material for: The links between supplementary tannin levels and conjugated linoleic acid (CLA) formation in ruminants: A systematic review and meta-analysis
Source: PLoS One. 2020 Mar 13;15(3):e0216187. doi: 10.1371/journal.pone.0216187 (PMC7069617; doi:10.1371/journal.pone.0216187)
Supplement: S1 Table — (DOCX) [file pone.0216187.s001.docx]

**S1A Table: Full electronic search strategy for ISI Web of Knowledge.**

| **Search** | **Search query** | **Items found** |
| --- | --- | --- |
| #1 | Biohydrogenation [Topic] | 1380 |
| #2 | Conjugated linoleic acid [Topic] | 8791 |
| #3 | Rumen [Topic] | 20671 |
| #4 | Tannin [Topic] | 22637 |
| #5 | Condensed tannin [Topic] | 4997 |
| #6 | Hydrolysable tannin [Topic] | 2641 |
| #7 | Meat [Title/Abstract] | 26614 |
| #8 | Milk [Title/Abstract] | 53500 |
| #9 | *In vivo* [Title/Abstract] | 143213 |
| #10 | *In vitro* [Title/Abstract] | 204802 |
| #11 | #1 OR #2 | 9298 |
| #12 | #1 AND #3 | 928 |
| #13 | #1 AND #3 AND #4 | 77 |
| #14 | #2 AND #3 AND #4 | 52 |
| #15 | #2 AND #3 AND #4 AND #5 OR #6 | 2672 |
| #16 | #2 AND #3 AND #4 AND #5 OR #6 AND #7 AND #9 OR #10 | 204832 |
| #17 | #2 AND #3 AND #4 AND #5 OR #6 AND #8 AND #9 OR #10 | 204832 |
| #18 | #2 AND #3 AND #4 AND #5 OR #6 AND #7 AND #8 AND #9 OR #10 | 204832 |
| #19 | #1 AND #3 AND #4 AND #5 OR #6 AND #7 AND #8 AND #9 OR #10 | 204840 |
| #20 | #1 AND #2 AND #3 AND #4 AND #5 OR #6 AND #7 AND #8 AND #9 OR #10 | 204822 |

**S1B Table: Full electronic search strategy for Mendeley.**

| **Search** | **Search query** | **Items found** |
| --- | --- | --- |
| #1 | Biohydrogenation | 1180 |
| #2 | Conjugated linoleic acid | 6672 |
| #3 | Rumen | 33781 |
| #4 | Tannin | 14954 |
| #5 | Condensed tannin | 2444 |
| #6 | Hydrolysable tannin | 464 |
| #7 | Meat | 144574 |
| #8 | Milk | 283876 |
| #9 | *In vivo* | 1215559 |
| #10 | *In vitro* | 1627361 |
| #11 | #1 OR #2 | 6862 |
| #12 | #1 AND #3 | 740 |
| #13 | #1 AND #3 AND #4 | 26 |
| #14 | #2 AND #3 AND #4 | 12 |
| #15 | #2 AND #3 AND #4 AND #5 OR #6 | 6 |
| #16 | #2, #3, #4, #5, #6, #7, #9, #10 | 13 |
| #17 | #2, #3, #4, #5, #6, #8, #9, #10 | 13 |
| #18 | #2, #3, #4, #5, #6, #7, #8, #9, #10 | 6 |
| #19 | #1, #3, #4, #5, #6, #7, #8, #9, #10 | 3 |
| #20 | #1, #2, #3, #4, #5, #6, #7, #8, #9, #10 | 4 |

**S1C Table: Full electronic search strategy for Scopus.**

| **Search** | **Search query** | **Items found** |
| --- | --- | --- |
| #1 | Biohydrogenation [All fields] | 4559 |
| #2 | Conjugated linoleic acid [All fields] | 28624 |
| #3 | Rumen [All fields] | 64893 |
| #4 | Tannin [All fields] | 88392 |
| #5 | Condensed tannin [All fields] | 18919 |
| #6 | Hydrolysable tannin [All fields] | 9372 |
| #7 | Meat [Article title, abstract, keywords] | 111074 |
| #8 | Milk [Article title, abstract, keywords] | 177011 |
| #9 | *In vivo* [Article title, abstract, keywords] | 938584 |
| #10 | *In vitro* [Article title, abstract, keywords] | 1319131 |
| #11 | #1 OR #2 | 30182 |
| #12 | #1 AND #3 | 3409 |
| #13 | #1 AND #3 AND #4 | 501 |
| #14 | #2 AND #3 AND #4 | 460 |
| #15 | #2 AND #3 AND #4 AND #5 OR #6 | 305 |
| #16 | #2 AND #3 AND #4 AND #5 OR #6 AND #7 AND #9 OR #10 | 13 |
| #17 | #2 AND #3 AND #4 AND #5 OR #6 AND #8 AND #9 OR #10 | 20 |
| #18 | #2 AND #3 AND #4 AND #5 OR #6 AND #7 AND #8 AND #9 OR #10 | 8 |
| #19 | #1 AND #3 AND #4 AND #5 OR #6 AND #7 AND #8 AND #9 OR #10 | 9 |
| #20 | #1 AND #2 AND #3 AND #4 AND #5 OR #6 AND #7 AND #8 AND #9 OR #10 | 5 |

**S1D Table: Full electronic search strategy for PubMed.**

| **Search** | **Search query** | **Items found** |
| --- | --- | --- |
| #1 | Biohydrogenation [Text word] | 576 |
| #2 | Conjugated linoleic acid [Text word] | 2781 |
| #3 | Rumen [Text word] | 9528 |
| #4 | Tannin [Text word] | 2883 |
| #5 | Condensed tannin [Text word] | 429 |
| #6 | Hydrolysable tannin [Text word] | 67 |
| #7 | Meat [Title/Abstract] | 39226 |
| #8 | Milk [Title/Abstract] | 84859 |
| #9 | *In vivo* [Title/Abstract] | 709160 |
| #10 | *In vitro* [Title/Abstract] | 911115 |
| #11 | #1 OR #2 | 3172 |
| #12 | #1 AND #3 | 399 |
| #13 | #1 AND #3 AND #4 | 10 |
| #14 | #2 AND #3 AND #4 | 3 |
| #15 | #2 AND #3 AND #4 AND #5 OR #6 | 69 |
| #16 | #2 AND #3 AND #4 AND #5 OR #6 AND #7 AND #9 OR #10 | 934132 |
| #17 | #2 AND #3 AND #4 AND #5 OR #6 AND #8 AND #9 OR #10 | 911115 |
| #18 | #2 AND #3 AND #4 AND #5 OR #6 AND #7 AND #8 AND #9 OR #10 | 911115 |
| #19 | #1 AND #3 AND #4 AND #5 OR #6 AND #7 AND #8 AND #9 OR #10 | 911115 |
| #20 | #1 AND #2 AND #3 AND #4 AND #5 OR #6 AND #7 AND #8 AND #9 OR #10 | 911115 |

**S1E Table: Full electronic search strategy for Google Scholar.**

| **Search** | **Search query** | **Items found** |
| --- | --- | --- |
| #1 | Biohydrogenation | 13100 |
| #2 | Conjugated linoleic acid | 76000 |
| #3 | Rumen | 219000 |
| #4 | Tannin | 93600 |
| #5 | Condensed tannin | 29500 |
| #6 | Hydrolysable tannin | 17000 |
| #7 | Meat | 2570000 |
| #8 | Milk | 1650000 |
| #9 | *In vivo* | 738000 |
| #10 | *In vitro* | 722000 |
| #11 | #1 OR #2 | 8660 |
| #12 | #1 AND #3 | 12100 |
| #13 | #1 AND #3 AND #4 | 1750 |
| #14 | #2 AND #3 AND #4 | 2040 |
| #15 | #2 AND #3 AND #4 AND #5 OR #6 | 1110 |
| #16 | #2 AND #3 AND #4 AND #5 OR #6 AND #7 AND #9 OR #10 | 768 |
| #17 | #2 AND #3 AND #4 AND #5 OR #6 AND #8 AND #9 OR #10 | 847 |
| #18 | #2 AND #3 AND #4 AND #5 OR #6 AND #7 AND #8 AND #9 OR #10 | 740 |
| #19 | #1 AND #3 AND #4 AND #5 OR #6 AND #7 AND #8 AND #9 OR #10 | 841 |
| #20 | #1 AND #2 AND #3 AND #4 AND #5 OR #6 AND #7 AND #8 AND #9 OR #10 | 496 |
