# Supplementary figures and images for: The links between supplementary tannin levels and conjugated linoleic acid (CLA) formation in ruminants: A systematic review and meta-analysis
Source: PLoS One. 2020 Mar 13;15(3):e0216187. doi: 10.1371/journal.pone.0216187 (PMC7069617; doi:10.1371/journal.pone.0216187)

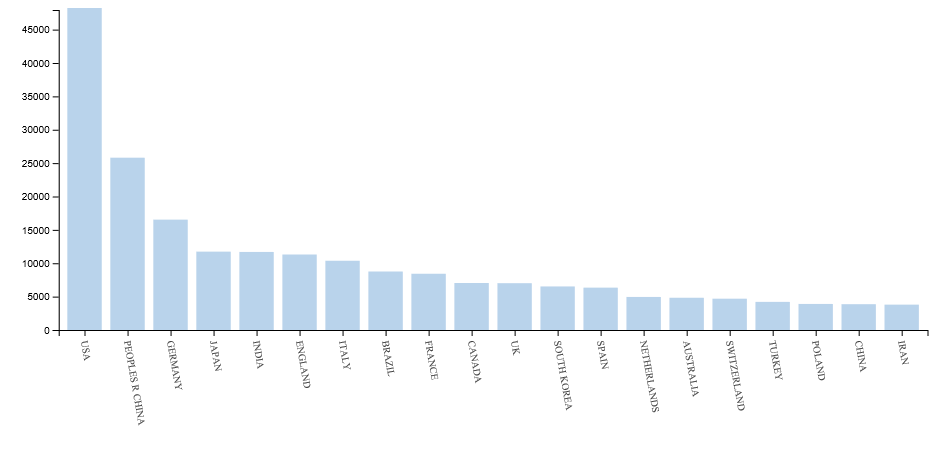

Supplement: S1 Fig — The databases were searched from January 1992 to March 2019. (TIF) [file pone.0216187.s004.tif]
